# Narrative exposure therapy in early intervention in psychosis services (NETp): protocol of a multi-site feasibility randomised controlled trial study

**DOI:** 10.1136/bmjopen-2026-121914

**Published:** 2026-06-22

**Authors:** Miriam Fornells-Ambrojo, James Plaistow, Amy Hardy, Peter Martin, Natasha Lyons, Zarina Khan, Adeliede Mberi, Tiyi Morris, Sonia Johnson

**Affiliations:** 1 Research Department of Clinical, Educational and Health Psychology, University College London, London, UK; 2 North London NHS Foundation Trust, London, UK; 3 Cambridgeshire and Peterborough NHS Foundation Trust, Fulbourn, UK; 4 Department of Psychology, King’s College London Institute of Psychiatry Psychology & Neuroscience, London, UK; 5 South London & Maudsley NHS Foundation Trust, London, UK; 6 Institute of Epidemiology and Health Care, University College London, London, UK; 7 Division of Psychiatry, University College London, London, UK; 8 Public representative, North East London NHS Foundation Trust, Rainham, UK; 9 Patient/Public Representative, Cambridgeshire and Peterborough NHS Foundation Trust, Fulbourn, UK; 10 Research Department of Clinical, University College London, London, UK

**Keywords:** Psychosocial Intervention, Clinical Trial, Schizophrenia & psychotic disorders, Randomized Controlled Trial, Stress Disorders, Traumatic, Acute

## Abstract

**Introduction:**

Cumulative interpersonal traumatisation increases the risk of psychosis and is linked to psychotic symptom severity and reduced recovery rates. Psychosis and treatment themselves can also be traumatising, with a first episode of psychosis posing challenges to one’s identity. Despite a growing evidence base for the safety and effectiveness of trauma therapies for psychosis, implementation remains poor in early intervention for psychosis (EIP) services, partly due to resource constraints. Narrative exposure therapy (NET) is an effective intervention for post-traumatic stress disorder in ethnically diverse populations. Crucially it has potential to address barriers to implementation of trauma therapies in EIP as it has a brief duration with minimal training requirements. This feasibility study will evaluate the feasibility and acceptability of conducting a definite trial of NET in people with early psychosis.

**Methods and analysis:**

An individually randomised parallel groups feasibility randomised controlled trial with embedded qualitative evaluation will be conducted. 50 individuals with first episode psychosis and a history of multiple trauma under the care of an EIP service in two NHS trusts in England will be recruited. Participants will be randomised to receive either 15 sessions of NET in addition to treatment as usual or treatment as usual alone. Assessor blinded assessments will be conducted at baseline, 4 months (post-intervention) and 8 months (follow-up). Analyses will focus on feasibility outcomes (recruitment, retention and completion rates) and preliminary estimates of intervention effects to inform planning for a fully powered trial. Qualitative interviews will be carried out with participants allocated to NET and clinicians to investigate acceptability, barriers and facilitators, possible mechanisms of change, potential benefits or harms and recommendations. A sub-study in the NET group will investigate acceptability of completing Experience Sampling Data daily during therapy to monitor changes on candidate primary and secondary outcomes.

**Ethics and dissemination:**

This study received REC/HRA ethical approval from the London - City & East Research Ethics Committee (REC) (25/LO/0350). The results of the study will be reported and disseminated at international conferences and in open-access peer-reviewed scientific journals.

**Trial registration number:**

ISRCTN39471182.

**STRENGTHS AND LIMITATIONS OF THIS STUDY**

- This multi-site randomised controlled trial with two follow-ups is set up in the national health service with local clinicians delivering narrative exposure therapy (NET). 
- Patient and public involvement embedded in all aspects of the study, alongside liberation psychology consultation, enhance ecological validity of assessments and therapy delivery.  
- The study uses mixed methods (questionnaires, qualitative interviews, experience sampling methodology) to evaluate feasibility which will inform refinements of the method and therapy protocol.
- An active control condition was not used, so it will not be possible to ascertain which elements of NETp produce change.

## Introduction

### Background

 Repeated trauma is very common in people with first episode of psychosis. Prevalence reports are around 80%, with about half also reporting symptoms of post-traumatic stress (42%).^1 2^ Cumulative childhood interpersonal traumatisation increases the risk of psychosis and is linked to greater severity of psychosis symptoms and reduced recovery rates.^3 4^ Psychosis and its treatment can also be traumatising, especially in the early stages of the condition, with testimonies suggesting frightening first encounters with symptoms such as abusive auditory hallucinations and coercive interventions.^5^ The latter is particularly true for minoritised groups who are disproportionately more likely to experience involuntary hospitalisation.^6^,^8^

Early intervention for psychosis (EIP) services are available internationally to people experiencing a first episode of psychosis and maintain contact with them for 3 years, aiming to improve prognosis by care in a timely manner to improve recovery rates. In England, the National Institute of Clinical Excellence (NICE)^9^ recommends that EIP should routinely assess for trauma and offer evidence-based psychological interventions for Post-Traumatic Stress Disorder (PTSD), and that these should be accessible for people from ethnic minorities. In practice, trauma interventions are not routinely offered to people in EIP, partly because of the scarcity of trauma-adapted protocols, training, and clinician safety concerns.^10^ NICE also recognises the need to further evaluate the efficacy and acceptability of trauma-focused therapies in EIP.

Trauma-focused interventions have a robust evidence base for adults with PTSD in the general population, with meta-analytic data showing large effect sizes for PTSD symptoms.^11^ However, psychosis has long been an exclusion criterion in trauma-focused intervention trials for PTSD, as it was assumed that this group might deteriorate with trauma treatment. We know now that trauma interventions for people with psychosis are safe and do not lead to iatrogenic harm.^12^ Recent reviews and meta-analyses of psychological interventions for trauma in individuals with psychosis reveal reductions in PTSD symptoms, particularly when employing exposure^13^; with improvements in psychotic symptoms identified specifically for delusions.^14^ However, lengthy treatments and complex training requirements are raised as implementation challenges, particularly in time-limited EIP services.^15^

Narrative exposure therapy (NET)^16^ is the only NICE-recommended intervention for PTSD that is specifically designed to address cumulative trauma by taking a longitudinal perspective, aiming to ameliorate PTSD symptoms but also to address the impact of repeated trauma on one’s personal narrative. This is important because after a first episode of psychosis, redefining one’s identity and finding meaning are key in the personal recovery journey.^5^

NET is based on psychological models of PTSD and memory^17^ but is also a testimonial therapy.^18^ NET aims to contextualise trauma memories, locating the threat and distress in the past rather than the here-and-now. After assessment and psychoeducation, there is a co-construction of the person’s lifeline, using physical materials offering an overview of the person’s life. Events are then narrated to integrate sensory-perceptual information with time, place and context, reducing the current threat. NET, initially proposed as a brief intervention for refugee populations, has been adapted to a range of client groups, and for online delivery.^19^ Meta-analytic evidence including 16 studies from both lower and higher income countries revealed large PTSD symptom reduction at post-treatment (g=1.18) and follow-up (g=1.37),^20^ high acceptability and particularly low dropout rates for NET in ethnically diverse populations, compared with other trauma-focused treatments.^20^ This is important, in light of persisting inequalities in the receipt of guideline recommended psychological interventions such as cognitive–behavioural therapy for psychosis (CBTp) in EIP services in England, with recent data showing that, compared with White British people, minoritized ethnic groups had lower odds of receiving CBTp by 20–61%.^21^

NET has potential to scale in the NHS and other healthcare systems as it is a short intervention with minimum training requirements (2–3 days) that has been effectively delivered by lay counsellors in developing countries.^22^ Effective delivery by a wider workforce is appealing in the context of NHS recruitment and retention challenges.^23^ In Western health systems, the broader suitability criterion for NET therapist training increases the possibility of expanding the workforce beyond just psychological professionals to include nurses and occupational therapists. This is in contrast to trauma-focused CBT that relies on scarce highly trained practitioners.^24 25^

Research on NET in psychosis is in its infancy. NET in psychosis case studies describe improvements in PTSD symptoms and paranoia.^26^ In 2021, Mauritz and colleagues^27^ reported a decrease in PTSD and psychotic symptoms in a Dutch single-group repeated measured study on 23 people with severe and enduring mental health difficulties (of whom 4 (17%) had a schizophrenia spectrum diagnosis), with a 0% dropout rate. No study to date has evaluated the feasibility, safety, acceptability and effectiveness of NET in EIP.

Our research group conducted preliminary qualitative studies exploring the experiences of NET in EIP in England with service users, carers, NET therapists and professionals.^28 29^ Participants spoke about the value of understanding the onset of psychosis, facing trauma and making sense of paranoia and voices in relation to one’s life to help prevent relapse. The reported benefits of NET included building a coherent life story, reductions in intrusive traumatic memories and self-blame. Recommendations were made for embedding NET in EIP, which was viewed as an ideal setting for offering NET, given the whole person approach, close working with families and carers and multidisciplinary working, where the range of health and social needs can be addressed in parallel.

### Objectives

The main objective is to evaluate the feasibility and acceptability of conducting a definitive trial of NETp in people with early psychosis. Specifically, the study will:

1. Assess whether n=50 people in EIP services are willing to be recruited to a trial and randomised to NET or Treatment as Usual (TAU).
2. Assess the acceptability of NET in EIP.
3. Assess the acceptability of the proposed outcome measures.
4. Describe the fidelity of intervention delivery.
5. Obtain preliminary estimates of effect sizes for NETp compared with TAU.
6. Obtain indicative estimates of other statistical quantities relevant for planning a fully powered trial (such as SD and therapists’ effects).

A pilot randomised controlled trial will be used to determine a range of parameters in relation to recruitment, assessment and therapy retention. Acceptability will be evaluated by qualitative interviews with clinicians and service users. Table 1 summarises the study objectives and their associated feasibility parameters.

**Table 1:** NETp trial feasibility outcomes and success criteria

Outcome	Criterion
Recruitment feasibility Number of eligible referrals received, willingness to consent and be randomised	80% Between 60% and 80% or Less than 60% of the target sample during the 10-month recruitment period
Assessment retention Number of participants who are lost to end-of-treatment assessment (4 months) and follow-up assessment (8 months) points	80% Between 60% and 80% or Less than 60% of participants complete the primary outcome measures at end-of-treatment (4 months) and follow-up assessments (8 months)
Therapy engagement (NET only group)* Intervention-specific data will include therapy engagement and number of sessions attended	80% Between 60% and 80% or Less than 60% of participants in the intervention arm attend at least 8 out of the 15 NET sessions
Therapy acceptability (NET only group)* Qualitative data with people with lived experience who receive NET and professionals shows that NET is acceptable and helpful	
Acceptability of measures Baseline, 4 months and 8 months assessments Session-by-session outcome monitoring data (NET group only) Acceptability of collecting daily Experience Sampling Method data during therapy (NET group only) Non-response rates to questionnaire(s) and items Assessments are rated as helpful in the Feedback about Measures Tool (FaM)	

* NET group only; A traffic light system will be adopted for the quantitative data. The intervention will be deemed either: (i) feasible to continue to a main trial, (ii) feasible but with modifications/ but requires further consultation with funders or (iii) not feasible.

NET, narrative exposure therapy.

## Methods and analysis

### Trial design and flow diagram

**Figure 1:**
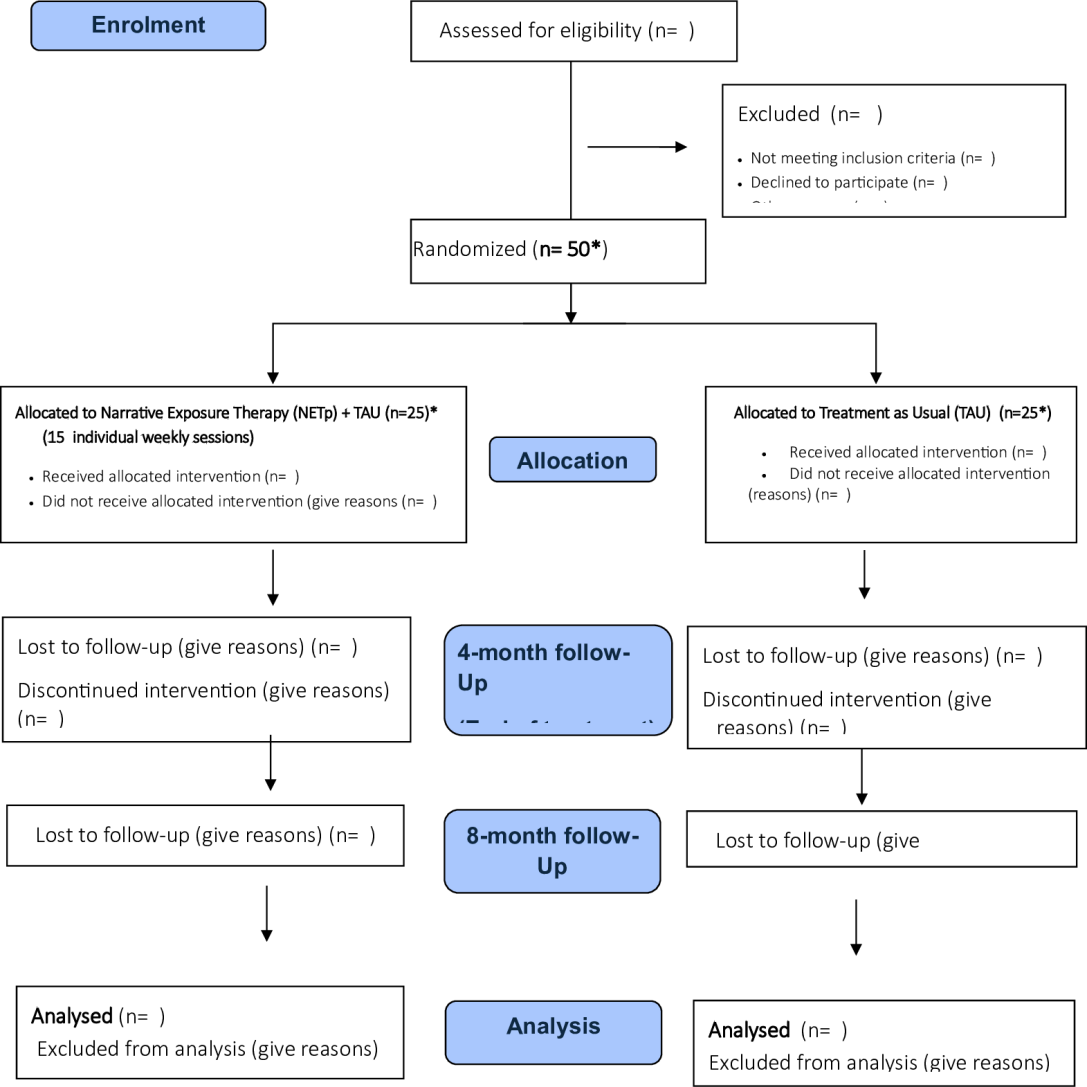
Consort flow diagram narrative exposure therapy (NET) in early intervention in psychosis (EIP). *Includes recruitment targets.

The NETp trial (‘Narrative Exposure Therapy in Early Intervention in Psychosis: A feasibility Randomised Control Trial (RCT) study’, ISRCTN39471182) is an individually randomised parallel groups feasibility RCT with embedded qualitative component. Participants will be randomly allocated to either NET plus TAU or TAU control. Please see the Consolidated Standards of Reporting Trial (CONSORT) flow diagram and schedule of participant activities in figure 1.

### Randomisation and blinding

Participants will be randomised to either NET or TAU, stratified by site and ethnicity (ethnic minority vs White British) using randomly permuted blocks—with block size randomised to 2 or 4. The randomisation sequence will be generated from random numbers generated in the R software for statistical computing^30^ and pre-loaded into the REDCap data management platform.^31 32^ The randomisation sequence will be stored but concealed within the REDCap data capture system. Research workers who complete baseline and follow-up assessment will be blinded to group allocation. Breaches in blindness will be recorded and, where feasible, assessments will be conducted by another blinded research worker.

### Participants

50 individuals will be recruited with first episode psychosis and a history of multiple traumas under the care of an EIP service in two NHS trusts: Cambridge and Peterborough NHS Foundation Trust (CPFT) and North London Mental Health Foundation Trust (NLFT). Purposive sampling will be undertaken to ensure that the sample is representative of the service user population. We will aim to recruit at least 20 of the 50 participants (40%) from minoritised ethnicities (ie, Black, Asian and mixed-race ethnic minority backgrounds (Ethnic minority background will be defined as all ethnic groups apart from White British)) to represent overall caseload people in the recruitment sites NLFT and CPFT (56% and 25%, respectively, from racialised groups).

### Inclusion criteria

- Report a history of multiple trauma (ie, more than one event in the Trauma and Life Events (TALE) checklist^33^ that ended at least a month ago and that still affects them (operationalised as TALE 21c item≥5).
- Report intrusive trauma symptoms as described below in (b.1) and/or (b.2):
  - (b.1) At least subthreshold post-traumatic symptoms in the previous week (ie, score of at least 2 on items 1–5) in the PTSD Checklist for Diagnostic and Statistical Manual of Mental Disorders, Fifth Edition (DSM-5) (PCL-5)^34^ and/or
  - (b.2) Current distressing symptoms of psychosis (ie, a score of 2 or above on the intensity of distress in the Psychotic Symptom Rating Scales (PSYRATS) Delusions/Hallucinations)^35^ or in the Adapted PSYRATS for Hallucinations in Other Modalities^36^ that are thematically linked to trauma (identified using an adapted version of section C of the Trauma Voices Association Questionnaire) and reporting a score>0.^37^
- On the caseload of an EIP (Please note additional diagnostic exclusion criteria preventing acceptance to the caseload of EIP services also include: Psychosis primarily caused by an organic condition; primary diagnosis of affective disorder without the presence of psychotic symptoms) service.
- Judged by the EIP care coordinator as clinically stable.
- Aged at least 18 years.
- Have capacity to give consent at the time of recruitment.

#### Exclusion criteria

- Primary diagnosis of substance/alcohol dependence, intellectual disability or cognitive dysfunction.
- Received a trauma-focused intervention from a qualified therapist for PTSD within the past 3 months.
- Insufficient English to provide informed consent or complete assessments without help from an interpreter.
- Lack capacity to consent.

### Assessments/outcomes

This trial is designed to evaluate the feasibility of conducting a future definitive trial. Therefore, the data collected at baseline, 4-month and 8-month follow-up assessments are intended to evaluate the feasibility of completing the battery of measures to be employed in the future trial.

### Eligibility

Table 2 shows the schedule of assessments. Informed consent (Please see online supplemental materials) will be sought by trained research assistants (RAs) prior to confirming eligibility criteria. The eligibility measures include the TALE checklist,^33^ the PTSD Checklist for DSM-5 (PCL-5),^34^ items from Questionnaire of Psychotic Experiences to identify the presence of hallucinations and distressing beliefs,^36^ the PSYRATS Delusions & Hallucinations^35^ and the adapted PSYRATS for Hallucinations in Other Modalities^36^ to evaluate the severity of psychotic experiences, and the adapted version of section C of the Trauma Voice Association Questionnaire^37^ to assess beliefs about associations between trauma and anomalous experiences. If screening criteria are met, the researcher will continue completing baseline measures.

**Table 2:** NETp trial schedule of enrolment, interventions and assessments

TIMEPOINT	Study period				
	Enrolment	Allocation		Post-trial allocation	Close-out
	−t_1_	t_0_ Baseline (pre-therapy)	Intervention	t_1_ (4 months, end of therapy assessment)	t_2_ (8 months follow-up, final assessment)
ENROLMENT					
Approach from clinical teams	X				
Informed consent for eligibility screen and participation	X				
Eligibility screen	X				
Allocation		X			
INTERVENTIONS					
*Narrative Exposure Therapy+TAU*			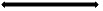		
*TAU*			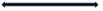		
ASSESSMENTS					
*Eligibility measures:* Trauma and life events checklist: (TALE) PTSD symptoms: (PCL-5) Current distressing psychotic symptoms present (QPE) and thematically linked to trauma screen: PSYRATS Delusions, Hallucinations/Hallucinations in other modalities and adapted TVAQ (Part C)	X				
***Demographic information***: Age, gender, ethnicity, religion, employment status, relationship status, education level, time since first episode of psychosis, duration of EIP support, psychiatric hospitalisations, current prescribed medication for mental health difficulties (including dosage), diagnosis		X			
***Outcome variables*** *Trauma and PTSD related measures:* PTSD Checklist for DSM-5 (PCL-5) The International Trauma Questionnaire (ITQ) Shutdown Dissociation Scale (Shut-D)		X (PCL-5 already completed as part of screen)		X	X
*Psychosis related measures:* PSYRATS Delusions, Hallucinations/Hallucinations in other modalities Green *et al* paranoid thought scales (R-GPTS)		X (PSYRATS already completed as part of screen)		X	X
*Other measures:* The Questionnaire about the Process of Recovery (QPR) Event Related Brief Shame and Guilt Scale (ERB-SGS) Depression Anxiety Stress Scales (DASS-21) Awareness of Narrative Identity Questionnaire (ANIQ) Feedback about Measures (FaM)		X		X	X
*Health Economics measures* EQ-5D-5L and CSRI		X		X	X
*Session-by-session measures being considered (NET group only*) ESM daily in between sessions to monitor progress (PTSD intrusions, psychosis symptoms, hope, context) Weekly monitoring at NET therapy session, items as in ESM+risk monitoring, cultural acceptability and bond) Feedback about Measures (FaM) – ESM and weekly			X X	x	
Qualitative interviews (*NET group only*) *Lived experience experts (n=25*) *NET clinicians (n=17*)				X (clinicians only after completing NET delivery)	X (service users only after completing NET delivery) and last follow-up)

EuroQol-5 Dimension, 5-Level questionnair (EQ-5D-5L (Herdman et al., 2011))

ANIQ, Awareness of Narrative Identity Questionnaire (Balzen et al., 2024); CSRI, Client Service Receipt Inventory (Beecham & Knapp, 2001)^46^; DASS-21v, Depression Anxiety Stress Scales (Brown et al., 1997)^43^; ERB-SGS, Event Related Brief Shame and Guilt Scale (Erb et al. 2023)^42^; ESM, Experience Sampling Methodology; FaM, Feedback about Measures (Fornells-Ambrojo et al., 2017)^48^; GPTS-R, Green Paranoid Thoughts Scale-Revised (Freeman et al., 2021)^41^; ITQ, International Trauma Questionnaire (Cloitre et al., 2018)^38^; PCL-5, The PTSD Checklist for DSM-5 (Blevins et al., 2015)^34^; PSYRATS, The Psychotic Symptoms Rating Scales (Haddock et al,1999)^34^; PSYRATS- HOMS, Adapted PSYRATS for Hallucinations in Other Modalities (Peters et al., 2022)^36^; QPE, Questionnaire of Psychotic Experiences (Rossell et al., 2019)^59^; QPR, The Questionnaire about the Process of Recovery (Neil et al., 2009)^40^; TALE, The Trauma and Life Events checklist (Carr et al., 2018)^33^; TVAQ, Trauma voice association questionnaire (van der Berg et al., 2022).

#### Primary outcomes

We specify candidate primary outcomes in this feasibility trial with a view to assessing which of them best meet the criteria for being primary outcomes in a full trial. This includes two measures of PTSD: PTSD assessed with the PCL-5^34^ and complex PTSD with the International Trauma Questionnaire (ITQ) (which includes assessment of PTSD and Disturbances of Self-Organisation)^38^ symptoms, and measures of psychosis impact assessed by the PSYRATS voices, distressing beliefs and hallucinations in Other Modalities.^35 36^

#### Secondary outcomes

Candidate secondary outcomes include PTSD related variables: PTSD/complex PTSD caseness (meeting diagnostic criteria according to the ITQ), severity of disturbances in self-organisation (complex PTSD subscale in the ITQ)^38^ and Dissociation (Shutdown Dissociation Scale; Shut-D)^39^; psychosis related measures, including a service user-defined measure of recovery (The Questionnaire about the Process of Recovery)^40^ and self-reported paranoia (The revised Green *et al* paranoid thought scales)^41^; in addition to guilt and shame (12-item Event Related Brief Shame and Guilt Scale),^42^ emotional distress (Depression Anxiety Stress Scales)^43^ and narrative identity (Awareness of Narrative Identity Questionnaire).^44^ To inform future economic analysis, the EQ-5D-5L^45^ tool to measure quality of life adjusted years (QALYs) and the Client Service Receipt Inventory (CSRI)^46^ to gather service use data will be included.

#### Experience sampling methodology (ESM)

m-Path is a mobile online platform designed to collect real-time data collection on experiences, emotions and behaviours. Participants allocated to the NET arm who consent to take part in this sub-study, will be prompted by a notification from the m-path app (https://m-path.io/landing/)^47^ once daily. Participants will receive an induction on the use of m-Path at baseline. This will involve personalisation (choosing a greeting/ending message), the time of the day they’d like to complete ESM data entry (a time between 08:00 and 22:00), and they will also be able to complete a practice questionnaire. Data completion is estimated to last approximately 2 min. Daily entering of data on a mobile app (https://m-path.io/landing/) will take place from 1-week pre-NET therapy (post randomisation) to 1 week post NET therapy completion. Areas covered include PTSD and cPTSD symptoms, unusual distressing experiences of psychosis, paranoia, mood, social connection, hope and context. m-Path procedures and items were co-produced. Participants will be made aware that their individual responses will be reviewed weekly by the clinician at the start of each of the 15 NET sessions, and by an unblinded researcher on the week after therapy ends.

#### Session-by-session monitoring

Participants allocated to NET will complete a weekly monitoring measure with the NET therapist. This will cover the same areas as in the ESM daily monitoring (that some participants will opt in to complete) in addition to three items enquiring about risk to self, cultural humility and therapeutic bond.

#### Acceptability of measures

Acceptability of measures will be assessed by non-response rates to items, and with the Feedback about Measures (FaM) tool.^48^ This will be completed in relation to primary and secondary outcome measure candidates at the end of baseline and follow-ups by all service user participants. Additionally, for those in the NET group, the FaM will also be completed at the end of therapy (i) by service user and clinician participants in relation to the session-by-session monitoring; and if agreed to complete ESM data, (ii) by service user participants at the last ESM data entry point, 1 week after the end of therapy, participants will be invited to provide feedback about their experience of completing ESM data on the m-Path app.

#### Qualitative interviews

Data collection and analysis will be guided by the Theoretical Framework of Acceptability^49^ using thematic analysis. The topic guides explore experiences of the NET intervention and its acceptability, barriers and facilitators, possible mechanisms of effect, potential benefits or harms and recommendations. Topic guides have been co-produced and developed with the study co-production group involving people with lived experience of psychosis and multiple trauma who receive care from EIP services, and also in consultation with Dr Taiwo Afuape, liberation psychology expert and trial collaborator, who advised on power imbalances and empowerment in the context of trauma.^50^

Service user participants who were offered NET (n=25) will be invited to complete a semi-structured interview after they complete the 8-month follow-up. Purposive sampling will be used to recruit a wide range of participants (eg, including people who dropped out if feasible). Interviews will usually be carried out by a person with lived experience, trained by the trial patient and public involvement (PPI) lead (NL), or if not available, interviews will be carried out by a Research Worker. NET therapists will also be interviewed by the Research Worker.

### Trial interventions

#### Narrative exposure therapy

NET is an evidence-based treatment for PTSD for multiple or prolonged traumatic events.^16^ Through detailed narration, traumatic memories are processed and contextualised, helping the person to develop a coherent autobiographical narrative. At the start of therapy, there is a co-construction of the person’s lifeline using physical materials, including traumatic events (represented by stones) and positive events (represented by flowers). Events are narrated in subsequent sessions in chronological order to ‘process’ the trauma memory and make meaning. We plan to offer 15 weekly individual sessions lasting 90 min. At the end of therapy, the person receives the written narrative as a documented testimony. The trial intervention will be provided as an addition to routine mental healthcare.

Face-to-face and online delivery will be offered. When online delivery is offered, e-NET guidance will be followed.^51^ Face-to-face therapy will be delivered at locations convenient for the participant resources permitting (eg, EIP team base, other NHS premises or residential locations) in line with the EIP assertive outreach model.^52^

The intervention will be delivered by a range of EIP professionals, including psychologists, CBT therapists, mental health nurses and occupational therapists (or professionals with equivalent experience, including those in the final year of training) within EIP. The manual for this feasibility trial will follow the standard NET manualised intervention,^16^ but it will also include specific guidance for addressing psychosis co-morbidity and delivery within an EIP service, in line with learning from pilot work and PPI consultation.^28 29^

NET clinicians will record information on session content, length, frequency, dates, number of sessions attended, number of sessions offered but not attended, mode (face to face/online) on a pre-defined database. All therapy sessions will be audio recorded if the participant gives written consent.

#### Treatment fidelity

NET training will take place over 2 days and will be provided by Katy Robjant, UK NET trainer, in collaboration with the NET Institute and Dr Maggie Schauer, one of the original developers of NET (https://www.net-institute.org). NET therapy fidelity will be monitored as follows: Treatment adherence will be monitored by asking therapists to complete a standardised session form to monitor session content after each therapy session that will be reviewed fortnightly during supervision sessions throughout the trial. Treatment competence will be assessed by a mixture of rating a random selection of therapy recordings (two sessions per therapist) using a NET fidelity scale developed by the NET Institute (Katy Robjant, UK NET trainer and project collaborator, personal communication, 2023).

#### Treatment as usual

This will be the routine care that participants receive including the EIP multi-disciplinary care from mental health nurses, psychiatrists, occupational therapists and psychologists in a community setting, with associated crisis and inpatient settings if required. EIP services offer up to 3 years of multidisciplinary support, including an allocated care coordinator and offer of antipsychotic medication and psychological interventions, such as CBTp and Family Intervention for psychosis.

### Adverse events

The occurrence of adverse events (AEs) will be monitored actively and systematically, following guidance from the CONSORT^53^ with the extension for social and psychological interventions^54^ and the extension for reporting of harms.^55^ AEs are defined as any untoward medical occurrence in a patient or trial participant, which does not necessarily have a causal relationship with the intervention involved. Each AE will be assessed for severity, seriousness, causality and expectedness as described below. Severity will reflect the impact of the events on the person at the time and have three levels (mild, moderate, severe). Seriousness relates to the outcome of the event, a serious adverse event (SAE) is defined as any AE that results in death, is life-threatening, requires hospitalisation or prolongation of existing hospitalisation, results in persistent or significant disability or incapacity or consists of a congenital anomaly or birth defect. No AEs or SAEs are expected as a result of the intervention. Temporary mild distress is expected to occur at the start of treatment and as part of exposure (eg, relieving the event) during trauma focus therapies. This is not predictive of poor treatment response in trauma-focussed therapies in people with psychosis.^56^

All AEs will be recorded in the Case Report Form (CRF) and sponsor’s AE log. SAEs will be reported to the sponsor within 24 hours of becoming aware. The CI (or a clinically qualified delegate) will review all SAE reports received. We will review the notes of all participants at the end of the trial to ensure thorough reporting.

We will report the number of AEs by trial arm and also give separate numbers of events by severity of the event and causality (related to the intervention, not related, not assessable).

### Data handling

The study will be compliant with the requirements of the General Data Protection Regulation (GDPR) (2016/679) and the UK Data Protection Act (2018). All investigators and study site staff will comply with the requirements of the GDPR (2016/679) with regard to the collection, storage, processing and disclosure of personal information and will uphold the Act’s core principles. University College London (UCL) is the data controller.

The CI will be the custodian for the trial data. The CRFs, paper questionnaires and online questionnaires will not bear the participant’s name or other personally identifiable data. All paper data will be anonymised and stored in a locked filing cabinet in the CI’s office at UCL (Research Department of Clinical, Health and Educational Psychology where the main Principal Investigator (PI) Dr Miriam Fornells-Ambrojo is based) or in the local NHS site in a location agreed by the local PI and local NHS Research and Development (R&D) team. The participant’s trial identification number will be used for identification. Anonymised data will be entered by RAs in the Research Electronic Data Capture system (REDcap) at University College London. Linked data (anonymised via participant ID) will be stored on a secure university electronic database for up to 10 years. Fully anonymised data will be kept indefinitely. Please see the study Data Management and Access Plan. Data will be deposited into the (Dataset) UCL Data Repository.

During the study, it is possible that a participant may disclose a serious risk of harm to themselves or to others. In these instances, participants’ confidentiality would be breached to share the necessary information with the relevant clinical team. The participant would be made aware of the limits of confidentiality in such instances in the Participant Information Sheet.

### Analysis

#### Quantitative analysis

Analyses will be carried out when the database has been cleaned and locked and after the statistical analysis plan has been finalised. We will use a CONSORT diagram to indicate the flow of participants through the study. We will report the numbers eligible, consenting and available for follow-up. We will document the recruitment pace and rate of consent to the study. We will report the number of participants meeting inclusion criteria via PTSD only, psychosis only and via both PTSD and psychosis routes. We will report the distribution of the number of NET sessions attended by each participant in the intervention arm, the proportion of participants who attend at least 8 out of the 15 NET sessions and therapy drop-out rates. The retention rate of participants as the proportion in each arm that provide responses at the 4-month and at the 8-month research assessment follow-ups will be reported alongside rates of item missingness (separately for each outcome measure). Furthermore, we will describe the distribution of responses on the FaM and conduct a qualitative thematic analysis to explore the responses to open-ended items. Treatment fidelity will be assessed by rating a random selection of therapy recordings (two sessions per therapist) using a NET fidelity scale developed by the NET Institute, with the distribution of the ratings described.

This study is a feasibility re and is not powered to evaluate evidence for the efficacy of NET vs TAU. Instead, we will report preliminary estimates that can inform sample size calculations for a future trial. All primary and secondary outcome measures are numeric variables and will be assessed at three time points. The 4-month follow-up will be the primary endpoint. Estimates of the treatment effect, NET versus TAU, at both follow-up points will be obtained from a multilevel model. Participants will be included in the outcome analysis if, for a given outcome, they have provided data at either the 4-month follow-up, the 8-month follow-up or both. The model can accommodate missing outcome values at any one of the two endpoints. Estimates are valid under the assumption that values are Missing at Random conditional on the baseline values and all outcome values included in the analysis. CIs will be estimated via a parametric bootstrap with 1000 bootstrap samples.

For all outcomes, we will report baseline and follow-up descriptive statistics. For the primary outcomes only, we will estimate the standardised effect sizes and therapist effects if feasible. Please see the further details in the Statistical Analysis Plan in online supplemental materials.

### Qualitative analysis

The qualitative interviews will be audio recorded, transcribed verbatim and analysed using Braun and Clarke’s thematic analysis.^57^ The aim is to understand participants’ experiences of receiving and providing the intervention, including its acceptability, safety and perceived usefulness. Analysis will be conducted on NVivo software. A critical realist perspective will be adopted, researcher reflexivity promoted and validity checks conducted. Service user interviews will be analysed first to give a primary voice to the experts by experience accounts.^58^ We will conduct credibility checks with participants. The theme structure will also be discussed with the NETp co-production group and research team. Please see further details in online supplemental materials.

### Economic analysis

For the early-stage economic analysis, the impact of the NET intervention on cost will be assessed compared with TAU. Health costs collected using the CSRI and QALYs (collected using the EQ-5D-5L) questionnaires will be compared at the baseline, 4-month and 8-month follow-up points and presented as mean values by study arm with SD. Unit costs from (available from the Personal Social Services Research Unit) and NHS Reference Costs will be used to generate NHS service costs. Lost work and informal care will be valued using average wage rates from the Office for National Statistics (ONS). Incremental QALYs and costs will be compared between each level of NET and routine care. In the case of one option having higher costs and producing more QALYs than another, we will present the incremental cost-effectiveness ratios to show the extra cost incurred to produce one more QALY. Uncertainty around the estimates will be explored using cost-effectiveness planes and acceptability curves.

### Patient and public involvement

Lived experience has been a core aspect of the project from the outset. We secured Public Involvement Fund (Ref: 5726) from the RDS East of England National Institute for Health and Care Research (NIHR) Research Design Service (RDS) to cover PPI costs during the application for NIHR funding. Two of the grant co-applicants are people with lived experience, including receipt of NET for PTSD while under the care of EIP. The Lived Experience NETp Co-Production Group, led by the trial PPI lead, includes service users under the care of EIP and has experienced multiple trauma and psychosis experiences and carers, with representation from minoritised backgrounds, a range of genders and ages. PPI has and will continue to shape all aspects of the study, from developing the trial and therapy protocols, selection of measures and procedures, shaping content of interview guides, conducting peer interviews, interpretation of findings and dissemination.

### Ethics and dissemination

This study was submitted to London - City & East Research Ethics Committee (REC) and has received REC/HRA ethical approval (25/LO/0350). Written informed consent will be gained from each participant before any study procedures are initiated. If amendments to the protocol are required, the same ethics committee will review them. UCL is the trial sponsor. (Email: uclh.randd@nhs.net). There are no trial criteria for discontinuing allocated interventions at the participant level, but participants will be made aware that if they find aspects of the research or therapy distressing and no longer want to continue, they will be able to withdraw without having to give a reason of this impacting on their routine clinical care.

The results of the study will be reported and disseminated at international conferences and in open-access peer-reviewed scientific journals. The results will also be available to participants and clinical teams in an accessible format and will be presented in a dissemination event. The study has a Trial Management Group overseeing the study, a Lived Experience NETp Co-Production Group as well as an independent Trial Steering Committee also supporting it.

### Trial status

Recruitment commenced in August 2025 (study protocol V1.0 date) and is planned to finish at the end of May 2026. Data collection is planned to be completed in January 2027.

## Supplementary material

10.1136/bmjopen-2026-121914 online supplemental file 1
