# Supplementary material for: Narrative exposure therapy in early intervention in psychosis services (NETp): protocol of a multi-site feasibility randomised controlled trial study
Source: BMJ Open. 2026 Jun 22;16(6):e121914. doi: 10.1136/bmjopen-2026-121914 (PMC13288681; doi:10.1136/bmjopen-2026-121914)
Supplement: online supplemental file 1 [file bmjopen-16-6-s001.pdf]

Supplementary Materials

1. Consent form NETp service user participants
2. Qualitative interview NET p (completers)
3. Qualitative interview NETp trial (non completers)
4. NETp SAP v1.1. 220526
5. Additional information on qualitative analysis

2. Consent form

CONSENT FORM

Narrative Exposure Therapy in Early Intervention in Psychosis:

A feasibility Randomised Control Trial (RCT) study v1.1; 02.05.25

IRAS ID: 337365 Name of Researcher:

Participant Identification Number for this trial:

Please initial box

1.	I confirm that I have read the information sheet dated 02.05.25 (version 1.1) for the above study. I have had the opportunity to consider the information, ask questions and have had these answered satisfactorily.	☐
2.	I understand that my participation is voluntary and that I am free to withdraw at any time without giving any reason, without my medical care or legal rights being affected.	☐
3.	I am willing for researchers to inform my clinical team that I am taking part in the project and provide information relevant to my care while I am taking part. I understand that this information will be added into my electronic notes.	☐
4.	I agree to my GP (General Practitioner) being informed of my participation in the study.	☐
5.	I allow the project team access to my clinical notes for information relevant to the trial. This access will remain for the duration of the trial.	☐
6.	I understand that information relating to me taking part in this study will be anonymised and stored on a secure university electronic database for up to 10 years. I understand that fully anonymised data will be kept indefinitely.	☐
7.	I understand that data collected during the study may be looked at by individuals from University College London, from regulatory authorities or from the NHS Trust, where it is relevant to my taking part in this research. I give permission for these individuals to have access to my data.	☐
8.	I know what to do if I have any concerns or want to make a complaint about any individual or the project.	☐
9.	I understand the potential risks of participating and the support that will be available to me should I become distressed during the course of the research. I understand the possible direct/indirect benefits of participating.	☐

When completed: 1 for participant; 1 for researcher site file; 1 to be kept in medical notes.

10. I understand that, if I withdraw from the study, the research team will continue to use the information ("data") they have about me already but will not contact any more about the study or collect any more data from me.	☐
11. I understand that the information I have submitted will be published as a report. All data will be anonymised, and the report will not include any personally identifying details.	☐
12. I agree that the information collected about me can be used to support other research in the future and may be shared anonymously with other researchers.	☐
13. *[ADDITIONAL Audio recording of NET therapy sessions to assess if NET therapist are delivering the treatment reliably and consistently, according to the NET model (this is known as treatment fidelity)]:* If I am allocated to receive NET, I agree for NET therapy sessions to be audio recorded for the purpose of evaluating the NET therapist fidelity to the NET model. Once NET fidelity has been rated by the NET consultant/supervisor the audio recording will be deleted.	☐
14. [ADDITIONAL Experience sampling check in between NET sessions]: I agree to take part in the experience-sampling part of the study if I receive Narrative Exposure Therapy, which involves completing brief questions in everyday life on a smartphone (for about 2 minutes) from the week before starting NET for the duration of the therapy to check how things are going (4 months).	☐
15. [ADDITIONAL Interview about NET experiences] I agree to take part in an interview about my experiences of NET therapy if I receive it during this study at 8 months. I understand that this interview would be recorded.	☐
16. *[ADDITIONAL Interview about NET experiences]* If I take part in an interview about my experiences of the therapy, I agree that the recording of my interview can be written up ("transcribed") by a UK-based, GDPR compliant transcription company.	☐
17. *[ADDITIONAL Interview about NET experiences]* If I take part in an interview about my experiences of the therapy, I give consent for anonymised sections to be taken from the audio recordings for use in written form in future presentation materials and scientific publications. I understand that these quotations will be anonymous.	☐
18. *[ADDITIONAL Future research]* I agree to be contacted by the research team in the future, I give permission for my contact details to be stored by the research team for this purpose.	☐
19. I understand that I will be offered £25 as a token of appreciation for each of the three meetings you complete with a researcher, up to a total of £75. Additionally, I understand that I am allocated to the NET arm of the study and I agree to take part in the two additional studies I will be offered: £60 for completing the daily in-between sessions Experience Sampling feedback on a mobile app at 4 months (at the end of the 15 NET sessions), and £25 for the interview about experiences of NET. I will not benefit financially in any other way from this study or from any possible outcome it may result in.	☐
20. *[OPTIONAL]* I would like to receive a summary of the overall study results once completed, and I consent to my contact details being kept for this purpose.	☐
21. I agree to take part in the above study.	☐

When completed: 1 for participant; 1 for researcher site file; 1 to be kept in medical notes.

Name of Participant	Date	Signature
Name of Person seeking consent	Date	Signature

When completed: 1 for participant; 1 for researcher site file; 1 to be kept in medical notes.

2.Qualitative interview NETp (completed)

Interview Schedule

VERSION FOR PARTICIPANTS WHO COMPLETED NET:

Your experience of receiving Narrative Exposure Therapy in a Early Intervention for Psychosis Service v1.0 10/12/24

Welcome!

Thank you so much for agreeing to meet with me today. How are you this morning/afternoon? (Informal chat).

My name is [interviewer], I'm one of the researchers [(if feel comfortable to disclose): with personal experience of using mental health services/early intervention services] working on the study.

Before we begin, I would first just like to re-cap a bit about what this conversation is about. So, the purpose of this research is to understand a bit more about what it is like doing sessions of Narrative Exposure Therapy for people in Early Intervention in Psychosis services. We hope the research will help us to understand how we can best support people with psychosis who have experienced traumatic events in their lives.

So I'd like to spend some time today hearing about your experience of the talking therapy named **Narrative Exposure Therapy- or NET that you received as part of the research trial** from (therapist name) that ended approximately (x months ago).

Just as a reminder, NET sessions start with constructing a lifeline of different life experiences *(show photo of generic lifeline)*.

- *(Prompt) Do you remember making a lifeline like this in therapy, using stones, flowers or other objects?*
- *(Prompt) Do you remember talking to (Name) about upsetting or traumatic events that have happened in your life?*
- *(Prompt) At the end of your therapy, do you remember if/when your therapist read out a testimony of all of the events you spoke about in therapy?*

We'll be focusing on how you found different aspects of the NET therapy. **I won't be asking any questions about the personal things you shared in therapy.** The questions will focus on what it was like having the NET sessions, any changes you noticed, and parts of the therapy you thought were helpful or unhelpful. There are no right or wrong answers- it's your own views that I'd like to hear about. What you share today won't have any impact on the care you receive in EIP.

The interview will last for up to one hour. You are free to stop the interview at any point and you do not have to answer any questions that you don't feel comfortable answering. Is there anything that you would like to check before we begin?

I have a voice recorder here that I'll be using to record the interview; it will also be recorded on Microsoft Teams. We haven't started recording yet, but I'll tell you when I'm about to start – is that OK? Just a reminder that the audio-recording will be stored on a secure computer drive until we have typed up the notes from the interview, and then it will be destroyed. No one outside the project will be allowed to access the recordings or interview notes and we will remove all identifying information from any reports.

Are you happy for me to start the interview? Okay, I will start the recording.

I'd like to start by asking some questions about when you started NET	
**1. Can you tell me about previous experience of psychology before NET** (if there have been any)?	*P: If yes, do you remember the name of the therapy and what was it for?*<br>*P: How did the previous therapy fit with NET? Was there some learning/ techniques from previous psychological intervention that were helpful when starting NET?*
**2. Why did you decide to take part in the study about Narrative Exposure Therapy?**	*P: Who's idea was it?*<br>*P: How did you feel about starting the therapy?*<br>*P: What did you want help with?*
**Thank you. The next few questions are about what it was like having sessions of NET**	
**3. How would you describe your overall experience of NET sessions?**	
**4. How did you feel when you were having the NET therapy sessions? What was it like talking to (therapist name)?**	*P. What was this like for you?*<br>*P. How would you describe the experience to another person thinking of having NET?*
**5. In your opinion, what were the challenges/burden/effort required for attending weekly NET sessions?**	*P. Were there any times that you wanted to stop the NET sessions?*<br>*p. If stopped: Please let us know Is there anything that could have been said or done to help you continue?*
**Thank you. The next questions ask if NET therapy addressed aspects of your culture and identity that are important for you. What we mean by identity is a certain characteristic that makes up and individual such as age, gender, ethnicity, sexuality, religion/spirituality, disability, relationship status, social class…What matters most to each person will vary from person to person.**	
**6. What aspect(s) of your identity are important to you?**	*P:* Some aspects that are important to people include, gender, ethnicity, sexuality, religion/spirituality, disability, relationship status, social class… are any of these are important to you?
**7. Were (**______________*please name the above aspect(s) of identity important to the person here*) **considered or discussed during NET? What was this like?**	*P: How did (therapist name) incorporate your identity in the sessions?*<br>*P: How did NET fit with your values?*
**8. Were (**______________*please name the above aspect(s) of identity important to the person here*) **not**	*P: Were there parts of the NET that didn't fit with aspects of who you are as a person?*

**considered or discussed during NET? What was this like?**	
**9. How did NET fit with your understanding of your experiences/difficulties?**	
**10. How did your culture (family, society) influence on your willingness/readiness to engage with NET? How did NET fit within your culture/cultural values?**	*P: Were there any concerns/ worries about engaging with NET linked to cultural values/ expectations? Please let me know what this was like for you.*
**Thank you for answering questions about identify and culture so far. The next question is about your views on how NET did in considering harmful or painful experiences you might have experienced in relation to your identity or culture. These experiences can include discrimination, marginalisation, persecution, oppression, and might be about yourself, family or your wider community.**	
**11. If you have experienced harmful or painful experiences in relation to your identity or culture, can you please tell me if and how NET addressed this during sessions?**	

**Thank you for answering those questions and sharing your experiences. Now I'm going to ask you some questions about what impact, if any, you feel the NET sessions might have had on you and your life.**	
**12. Can you tell me about any benefits you noticed from having the NET sessions (if there were any)? What was most important to you? How did it help (if it did)?**	*P. Was there anything that got better during the NET sessions?*<br>*P: Was NET helpful? If yes, how did it help you?*<br>*P. Were there any changes on how you felt/ the things you were able to do/how you see yourself and others/intrusive memories (e.g. flashbacks) or distressing anomalous experiences (e.g. voices, paranoia)? How did this change?*
**13. Can you tell me about anything you would have liked to have changed that hasn't changed after doing NET? Was there anything that got worse?**	*P:*
**14. If unusual distressing experiences like voices or paranoia were present while you did NET, what**	

**was this like for you? Did they affected your ability to work with the NET therapist? What was this like?**	
**15. If you are/were taking medication prescribed by the Early Intervention Psychosis Team (e.g. antipsychotic medication, antidepressants) while you had NET therapy…. What do you think the role of medication was in relation to any changes reported with NET?**	
**We want to understand if there are certain aspects of NET that are particularly helpful for people. I'm going to ask some questions about how you found parts of the therapy- this might be things the therapist said or did, things you and the therapist did together, or something about the relationship you had with the therapist.**	
**16. What, if any, was helpful about NET in addressing traumatic memories?**	*P. What about the NET sessions helped bring about any changes you were hoping for?*<br>*P. Were there any specific moments that you think helped change things?*
**17. What aspects of the therapy, if any, did you value or enjoy the most? Why was that?**	*P. What was that like for you?*
**18. Were there any aspects of the NET sessions that felt unhelpful? What happened? What was that like?**	*P. By unhelpful, perhaps anything that made yo[illegible] upset and felt unnecessary to making progress.*
**19. In your opinion, what was it like creating the lifeline (placing flowers and stones along a string) with the therapist? What did it mean to you?**	*P: How did you find placing using stones and flowers across the lifeline to represent traumas and positive events in your life?*
**20. How was it like hearing back the narrations of the events you have been through? (i.e. when the therapist wrote and then read back to you the narration of the previous session?**	
**Thank you for all the feedback that you have shared with me today. To finish off, I have a couple of questions about NET generally, and what it has been like talking about the NET sessions with me today.**	
**21. Is there anything else you would like to tell me about your experience of NET that we have not covered in the interview today?**	
**19. Are there any questions about NET that you think we should have included in this interview?**	
**22. How did this interview made you feel? How do you feel now?**	

We have come to the end of the interview. Thank you for your time and for sharing your experiences with me. I really appreciate it.

Ask participants if they are feeling upset, if they would like to go through the debrief sheet together and do breathing exercises.

Remind participants that after leaving the interview, if at any point they feel upset, worried or distressed they can contact their care coordinator or call the Samaritans 24/7.

Thanks again, I hope you have a good rest of the day.

3. Qualitative interview NETp (not completed)

Interview Schedule

VERSION FOR PARTICIPANTS WHO DID NOT COMPLETE NET:

Your experience of receiving Narrative Exposure Therapy in a Early Intervention for Psychosis Service v1.0 9/12/24

Welcome!

Thank you so much for agreeing to meet with me today. How are you this morning/afternoon? (Informal chat).

My name is [interviewer], I'm one of the researchers [(if feel comfortable to disclose): with personal experience of using mental health services/early intervention services] working on the study.

Before we begin, I would first just like to re-cap a bit about what this conversation is about. So, the purpose of this research is to understand a bit more about what it is like to be offered sessions of Narrative Exposure Therapy for people in Early Intervention in Psychosis services. We hope the research will help us to understand how we can best support people with psychosis who have experienced traumatic events in their lives.

We understand that Narrative Exposure Therapy is not for everybody and want to understand your experiences to help us understand how to support people in the future. You might have changed your mind and decided not to attend any NET session or maybe you attended some and then decided/ were unable to complete the intervention offered. Your experience is important to us.

So I'd like to spend some time today hearing about your views of the talking therapy named **Narrative Exposure Therapy- or NET that you were offered as part of the research trial** from (therapist name) that ended approximately (x months ago).

Just as a reminder, NET sessions start with constructing a lifeline of different life experiences *(show photo of generic lifeline)*. Following sessions involve talking about upsetting or traumatic experiences that the therapist writes down as testimony that is read back to you in following sessions.

Do you remember being offered NET? YES NO

Please tick the one that applies:

- ☐ I did not attend any NET sessions
- ☐ I attended the introductory NET session
- ☐ I attended up to the NET session where we did the lifeline
- ☐ I attended up to the NET sessions where I started narrating upsetting or traumatic experiences

We'll be focusing on your views on NET therapy. **I won't be asking any questions about the personal things you shared in therapy.** The questions will focus on what it was like being offered NET therapy and if you attended any session what that was like, what you thought was helpful or unhelpful. There are no right or wrong answers- it's your own views that I'd like to hear about. What you share today won't have any impact on the care you receive in EIP.

The interview will last for up to one hour. You are free to stop the interview at any point and you do not have to answer any questions that you don't feel comfortable answering. Is there anything that you would like to check before we begin?

I have a voice recorder here that I'll be using to record the interview; it will also be recorded on Microsoft Teams. We haven't started recording yet, but I'll tell you when I'm about to start – is that OK? Just a reminder that the audio-recording will be stored on a secure computer drive until we have typed up the notes from the interview, and then it will be destroyed. No one outside the project will be allowed to access the recordings or interview notes and we will remove all identifying information from any reports.

Are you happy for me to start the interview? Okay, I will start the recording

**I'd like to start by asking some questions about when you started NET**	
**1. Can you tell me about previous experience of psychology before NET (if there have been any)?**	*P: If yes, do you remember the name of the therapy and what was it for?*<br>*P: How did the previous therapy fit with NET? Was there some learning/ techniques from previous psychological intervention that were helpful when starting NET?*
**2. Why did you decide to take part in the study about Narrative Exposure Therapy?**	*P: Who's idea was it?*<br>*P: How did you feel about starting the therapy?*<br>*P: What did you want help with?*
**3. [ONLY ASK THIS QUESTION IF THE PARTICIPANT DID NOT ATTEND ANY NET SESSIONS, IF NOT PROCEED TO QUESTION 4]**<br>**If you did not attend any NET sessions after being randomly allocated to receiving them: Please let us know what influenced this decision?**	*P: We are keen to know if there was anything about the research process that had an impact*<br>*P: What there anything about how NET was explained to you that made you decide this therapy was not for you? What was it?*<br>*P: Did you have any worries or concerns about starting NET?*
**ONLY PROCEED TO QUESTIONS 4+ IF THE PARTICIPANT ATTENDED AT LEAST ONE NET SESSION**	
**Thank you. The next few questions are about what it was like attending the NET sessions you were able to attend**	
**3. How would you describe your overall experience of NET sessions?**	
**4. How did you feel when you were having the NET therapy sessions? What was it like talking to (therapist name)?**	*P. What was this like for you?*<br>*P. How would you describe the experience to another person thinking of having NET?*
**5. In your opinion, what were the challenges/burden/effort required for attending weekly NET sessions?**	*P. Were there any times that you wanted to stop the NET sessions?*<br>*p. If stopped: Please let us know Is there anything that could have been said or done to help you continue?*
**Thank you. The next questions ask if NET therapy addressed aspects of your culture and identity that are important for you. What we mean by identity is a certain characteristic that makes up and individual such as age, gender, ethnicity, sexuality, religion/spirituality, disability, relationship status, social class…What matters most to each person will vary from person to person.**	

**6. What aspect(s) of your identity are important to you?**	*P:* Some aspects that are important to people include, gender, ethnicity, sexuality, religion/spirituality, disability, relationship status, social class… are any of these are important to you?
**7. Were (____________** *please name the above aspect(s) of identity important to the person here*) **considered or discussed during NET? What was this like?**	*P: How did (therapist name) incorporate your identity in the sessions?*<br>*P: How did NET fit with your values?*
**8. Were (____________** *please name the above aspect(s) of identity important to the person here*) **not considered or discussed during NET? What was this like?**	*P: Were there parts of the NET that didn't fit with aspects of who you are as a person?*
**9. How did NET fit with your understanding of your experiences/difficulties?**	
**10. How did your culture (family, society) influence on your willingness/readiness to engage with NET? How did NET fit within your culture/cultural values?**	*P: Were there any concerns/ worries about engaging with NET linked to cultural values/ expectations? Please let me know what this was like for you.*
**Thank you for answering questions about identify and culture so far. The next question is about your views on how NET did in considering harmful or painful experiences you might have experienced in relation to your identity or culture. These experiences can include discrimination, marginalisation, persecution, oppression, and might be about yourself, family or your wider community.**	
**11. If you have experienced harmful or painful experiences in relation to your identity or culture, can you please tell me if and how NET addressed this during sessions?**	

**Thank you for answering those questions and sharing your experiences. Now I'm going to ask you some questions about what impact, if any, you feel the NET sessions might have had on you and your life.**	
**12. Can you tell me about any benefits you noticed from having the NET sessions (if there were any)? What was most important to you? How did it help (if it did)?**	*P. Was there anything that got better during the NET sessions?*<br>*P: Was NET helpful? If yes, how did it help you?*

	P. Were there any changes on how you felt/ the things you were able to do/how you see yourself and others/intrusive memories (e.g. flashbacks) or distressing anomalous experiences (e.g. voices, paranoia)? How did this change?
**13. Can you tell me about anything you would have liked to have changed that hasn't changed after doing NET? Was there anything that got worse?**	
**14. If unusual distressing experiences like voices or paranoia were present while you did NET, what was this like for you? Did they affected your ability to work with the NET therapist? What was this like?**	
**15. If you are/were taking medication prescribed by the Early Intervention Psychosis Team (e.g. antipsychotic medication, antidepressants) while you had NET therapy…. What do you think the role of medication was in relation to any changes reported with NET?**	*Prompt: If the participant did not attend many NET sessions/ they do not feel NET was helpful , interviewer can ask instead: What do you think it's the role of medication in*
**We want to understand if there are certain aspects of NET that are particularly helpful for people. I'm going to ask some questions about how you found parts of the therapy- this might be things the therapist said or did, things you and the therapist did together, or something about the relationship you had with the therapist.**	
**16. What, if any, was helpful about NET in addressing traumatic memories?**	*P. What about the NET sessions helped bring about any changes you were hoping for?*<br>*P. Were there any specific moments that you think helped change things?*
**17. What aspects of the therapy, if any, did you value or enjoy the most? Why was that?**	*P. What was that like for you?*
**18. Were there any aspects of the NET sessions that felt unhelpful? What happened? What was that like?**	*P. By unhelpful, perhaps anything that made you upset and felt unnecessary to making progress.*

**19. In your opinion, what was it like creating the lifeline (placing flowers and stones along a string) with the therapist? What did it mean to you?**	*P: How did you find placing using stones and flowers across the lifeline to represent traumas and positive events in your life?*
**20. How was it like hearing back the narrations of the events you have been through? (i.e. when the therapist wrote and then read back to you the narration of the previous session?**	
**Thank you for all the feedback that you have shared with me today. To finish off, I have a couple of questions about NET generally, and what it has been like talking about the NET sessions with me today.**	
**21. Is there anything else you would like to tell me about your experience of NET that we have not covered in the interview today?**	
**19. Are there any questions about NET that you think we should have included in this interview?**	
**22. How did this interview made you feel? How do you feel now?**	

We have come to the end of the interview. Thank you for your time and for sharing your experiences with me. I really appreciate it.

Ask participants if they are feeling upset, if they would like to go through the debrief sheet together and do breathing exercises.

Remind participants that after leaving the interview, if at any point they feel upset, worried or distressed they can contact their care coordinator or call the Samaritans 24/7.

Thanks again, I hope you have a good rest of the day.

Narrative Exposure Therapy for Psychosis (NETp)

Narrative Exposure Therapy in Early Intervention in Psychosis: A feasibility Randomised Control Trial (RCT) study

Statistical analysis plan

Written by: Peter Martin (Statistician)

Version 1.1, 22/05/2026

Version history log

Version	Date	Details of Change
1.0	4 Dec 2026	---
1.1	22 May 2026	Changes:<br>- Corrected the measurement of the primary outcome "Complex PTSD symptoms"<br>- Corrected the measurement of the secondary outcome "Severity of disturbances in self-organization"

Introduction

This analysis plan sets out the methods of analysing the predetermined primary and secondary outcomes of the NETp feasibility trial, as well as analyses to address the feasibility testing aims of the study. This analysis plan covers the statistical analysis of the clinical outcomes and feasibility indicators. It does not cover health economics, or qualitative data collection and analysis.

Further information on this study can be found in the protocol, version 1.0, 12 March 2025.

Aims

To assess

(1) the feasibility of delivering narrative exposure therapy in early intervention psychosis,
(2) the feasibility of evaluating this intervention in a randomized controlled trial.

Objectives

1. To assess whether people in early intervention psychosis (EIP) services are willing to be recruited to a trial and randomised to NET or Treatment as Usual (TaU)
2. To assess the acceptability of narrative exposure therapy in EIP
3. To assess the acceptability of the proposed outcome measures
4. To describe the fidelity of intervention delivery
5. To obtain preliminary estimates of the efficacy of NETp compared to TaU
6. To obtain indicative estimates of statistical quantities relevant for planning a fully powered randomized controlled trial

Study population

The study population comprises adults under the care of an early intervention for psychosis team who report a history of multiple trauma and current intrusive trauma experiences. They must be judged by the early intervention for psychosis care coordinator as clinically stable and be aged at least 18 years. Exclusion criteria are a primary diagnosis of substance/alcohol dependence, intellectual disability or cognitive dysfunction and the receipt of a trauma focussed intervention from a qualified therapist for PTSD within the past 3 months. See the protocol for further details.

Study design

This is a mixed-methods feasibility randomized controlled trial. The statistical analysis plan focuses on the statistical aspects. See the protocol for further details.

Intervention

Participants randomized to the intervention arm will receive 15 sessions of narrative exposure therapy of 90 minutes each. Participants in the control arm will receive treatment as usual.

Sample size

The study aims to recruit 50 patients in total. There was no formal sample size calculation.

Data collection

Service user characteristics

We will describe service user characteristics collected at screening and baseline: age, gender, ethnicity, employment status, relationship status, education level, time since first episode of psychosis, duration of EIP support, psychiatric hospitalizations, current prescribed medication for mental health difficulties (including dosage), diagnosis.

Outcomes

Outcome measures will be collected at screening/baseline, at 4 months after randomization, and at 8 months after randomization.

Primary outcomes

- Traumatic intrusions (The PTSD Checklist for DSM-5; PCL-5)
- Psychotic symptoms (The psychotic symptom rating scales for voices and distressing beliefs; PSYRATS – see the next section for details of coding these variables)
- Hallucinations in Other Modalities (adapted PSYRATS)
- Complex PTSD symptoms (PTSD subscale plus Severity of disturbances in self-organization (DSO) in the International Trauma Questionnaire; ITQ)

Secondary Outcomes

- Severity of disturbances in self-organization (DSO subscale subscale in the ITQ)

- PTSD/complex PTSD caseness (meeting diagnostic criteria according to the ITQ)
- A service user-defined measure of recovery (The Questionnaire about the Process of Recovery; QPR)
- Dissociation (Shutdown Dissociation Scale; Shut-D)
- Paranoia (The revised Green et al. paranoid thought scales (R-GPTS))
- Guilt and shame (12-item Event Related Brief Shame and Guilt Scale; ERB-SGS)
- Emotional distress (Depression Anxiety Stress Scales; DASS-21)
- Narrative identity (Awareness of Narrative Identity Questionnaire; ANIQ)
- Quality of life (EQ-5D-5L)

Detailed scoring instructions for all primary and secondary outcomes are given in the trial protocol, appendix 3.

Coding PSYRATS

Special coding procedures are pre-specified for the PSYRATS scales. These apply to (i) PSYRATS voices, (ii) PSYRATS other anomalous experiences, and (iii) PSYRATS distressing beliefs.

At all time points, prior to completing the PSYRATS scales, participants are asked about the presence of three symptom domains: (i) voices, (ii) anomalous experiences and (iii) distressing beliefs, using items from the Questionnaire of Psychotic Experiences (QPE). If a participant reports absence of a particular domain (e.g. hearing voices), the relevant PSYRATS scale (voices) will not be administered to that participant. At follow up points, participants will be asked again about each of the three PSYRATS domains in the QPE items, even if they indicated a particular symptom being absent at baseline. If a symptom is reported as present, the PSYRATS scale of that particular domain will then be administered at follow-up, even if absence of that domain was reported at baseline.

If a participant reports absence of a particular PSYRATS domain at all three time points, they will receive a score of "not applicable" for that PSYRATS domain at all three time points, and thus be excluded from the outcome analysis of that particular PSYRATS domain. If a participant reports presence of a particular PSYRATS domain at some time points, but reports absence of the same symptom domain at other time points, the participant shall receive a score of 0 for those time points where they reported absence of symptoms, and they will be included in the outcome analysis for that domain.

PSYRATS: exploratory composite measure

As an exploratory analysis, we shall calculate a composite measure of all three PSYRATS domains as the average of PSYRAT domains scores across all domains on which the participant gave a non-zero response. This composite measure will be analysed using the same outcome model as the PSYRAT domain scores.

Data entry and checking

Data will be entered using the REDCAP software. Before analysis and database lock, basic checks will be performed on the quality of the data, focusing on identifying:

- Missing data
- Data outside the expected range
- Other inconsistencies between variables e.g. in the dates the questionnaires were completed

If any inconsistencies are found, the corresponding values will be double checked with the researchers and corrected if necessary in the source data. This checking process and subsequent changes will be documented.

Statistical analyses

Analyses will be carried out when the database has been cleaned and locked and after the statistical analysis plan has been finalised.

We will use a consort diagram to indicate the flow of participants through the study. This will include the numbers eligible, the number consenting and the number available for follow up and will include reasons for non-consent and withdrawal and drop out. This will be constructed in conjunction with the study manager and research assistants.

Objective 1: To assess whether people in EIP services are willing to be recruited to a trial and randomised to NET or Treatment as Usual (TaU)

We will document the rate of consent to the study (number of patients consenting divided by number of patients eligible and invited), and the pace of recruitment (number of patients recruited per month over the duration of data collection).

We will report the number of participants meeting inclusion criteria via PTSD only, psychosis only, and via both PTSD and psychosis routes.

Objective 2: To assess the acceptability of NET in EIP

We will document the distribution of the number of NET sessions attended by each patient in the intervention arm, and report the proportion of patients who attend at least 8 out of the 15 NET sessions. We will also document study dropout rates separately for the TaU and NET arms of the study, and the treatment drop-out rate in the NET arm of the study.

Objective 3: To assess the acceptability of the proposed outcome measures

We will document the retention rate of participants as the proportion in each arm that provide responses at the 4-month follow-up and at the 8-month follow-up, respectively. We will also document the rates of item missingness among those participants that complete follow-up measures, separately for each outcome measure.

Furthermore, we will describe the distribution of responses to Question 1 in the Feedback about measures tool (FaM), separately for baseline assessment, the 4-months follow-up and the 8-months-follow-up. This item is rated on a scale from 0 – 10 (extremely unhelpful – extremely helpful). Qualitative thematic analysis will explore the responses to open-ended items 2 & 3 on this tool.

The FaM is also applied to session-by-session measures and experience sampling data collection. The distributions of responses to item 1 will be described separately for each of these.

Objective 4: To describe the fidelity of intervention delivery

Treatment fidelity will be assessed by rating a random selection of therapy recordings (2 sessions per therapist) using a NET fidelity scale developed by the NET Institute. The distribution of ratings will be described.

Objective 5: To obtain preliminary estimates of the efficacy of NETp compared to TaU

This study is a feasibility RCT and is not powered to evaluate evidence for the efficacy of NET versus TaU. The purpose of objective 5 is to obtain preliminary estimates that can inform sample size calculations for a future trial.

All primary and secondary outcome measures are numeric variables and will be assessed at three time points: baseline, 4-month follow-up, and 8-month follow-up. The 4-month follow-up will be the primary endpoint.

Estimates of the treatment effect, NET vs TaU, at both follow-up points will be obtained from a multilevel model of the form:

$$Y_{ti} = \beta_0 + u_i + \beta_1 X_i + \beta_2 X_i T_t + \beta_3 Y_{Bi} + \varepsilon_{ti},$$
$$u_i \sim N(0, \sigma_u^2);\ \varepsilon_{ti} \sim N(0, \sigma_\varepsilon^2)$$

Where:

- Y_{ti} is the outcome score at time t for the ith participant
- t = {0,1} identifies follow-up time points, where 0 denotes the 4-month follow-up and 1 denotes the 8-month follow-up
- i = {1, …, N} identifies participants, where N is the total sample size
- X_i is the treatment indicator (X = 0 for control arm, and X = 1 for intervention arm)
- T_t is a time indicator (T_0 = 0 for 4-month follow-up, and T_1 = 1 for 8-month follow-up)
- Y_{Bi} is the outcome variable score at baseline for individual i
- u_i is a random intercept for individual i.

The treatment effect at four-month follow-up will be estimated by the coefficient β_1, while the treatment effect at 8-month followup will be estimated as the sum of the coefficients $\beta_1 + \beta_2$. No further adjustments for covariates will be made, in light of the small sample size.

Participants will be included in the outcome analysis if, for a given outcome, they have provided data at either the 4-month follow-up, the 8-month follow-up, or both. The model can accommodate missing outcome values at any one of the two endpoints. Estimates are valid under the assumption that values are Missing at Random conditional on the baseline values and all outcome values included in the analysis.

Confidence intervals will be estimated via a parametric bootstrap with 1000 bootstrap samples.

Analysing PSYRATS scales:

We will report the number and proportion of participants reporting each type of psychotic symptom measured by the PSYRATS scaled separately for the two treatment arms. Otherwise PSYRATS scale will be analysed as outlined above.

Objective 6: To obtain indicative estimates of statistical quantities relevant for planning a fully powered randomized controlled trial.

For all outcomes:

- Baseline and follow-up descriptive statistics, including means, standard deviations, medians and interquartile ranges (separately for each treatment arm at all time points, pooled statistics at baseline)

For the primary outcomes only, we will estimate the following quantities:

- Standardized effect sizes: mean difference between treatment arms divided by the pooled baseline standard deviation, separately for each follow-up. Confidence intervals will be estimated via the parametric bootstrap with 1000 bootstrap samples.
- Therapist effects: We will attempt to estimate the intra-therapist correlation by re-estimating a modified outcome model separately for each follow-up time point, adding a random intercept for therapist. Due to the small sample size and the potentially large number of therapists, these models may not be identifiable. In that case, we will simply document the number of therapists.

Attrition and loss to follow-up

Patients who do not provide any follow-up data cannot be included in the analyses for objective 5 (outcome analyses), but will be included in other analyses as appropriate. Reasons for missing outcome data will be described. The frequency and percentage of subjects with missing data, by reason, will be provided for each randomised group (and for each outcome).

Missing items in scale measures

We will document the number and percentage of missing item values (see Objective 3). Missing items within summary scales will be prorated if item missingness is < 2 % in the sample as a whole, and fewer than 10 % of individuals have more than 1 item missing. If either total item missingness ≥ 2 % or at least 10 % of individuals have more than one item missing, we will conduct multiple imputation of missing values.

If prorating (mean imputation) is chosen, we will prorate scores for those individuals who have up to 20 % of items missing in a given outcome scale. For example, in a scale with 20 items, imputation will be applied to individuals with up to 4 items missing. The average value for the complete items will be calculated for that individual and used to replace the missing values. The scale score will be calculated based on the complete values and these replacements.

Exploratory analyses

Participants in the intervention arm will be invited to complete daily surveys of Experience Sampling Measures (ESM) on a smart phone app. We will document the rate of consent to this invitation, and the rate of completion of surveys, separately for each question. Exploratory analyses will investigate the quality of the data. Depending on the amount of ESM data are collected, we may conduct exploratory analyses of the trajectories of reported scores over time.

Adverse Events

The recording of adverse events is detailed in the study protocol, section 12. We will report the number of adverse events by trial arm, and also give separate numbers of events by severity of the event (mild, moderate, severe) and causality (related to the intervention, not related, not assessable).

5. Further details on qualitative analysis

Two researchers, trainee clinical psychologists Appey Ogenyi (AP) and Mawada Ghanem (MG), will lead on service user and clinician interviews respectively. For the service user interviews, we will aim for these to be carried out by a person with lived experience, trained by the trial PPI lead (NL), or if not available, interviews will be carried out by the trainee clinical psychologist AP. When interviews are carried out by a peer researcher with lived experience, trainee clinical psychologist AP will also be present during the interview. We are aiming for two people with lived experience (peer researchers) MB and ZK to conduct the service user interviews, but the final number of peer interviewers might vary depending on the lived experience researcher's preferences (e.g. for how many interviews they wish/feel able to complete), their availability, as well as research governance approvals for peer researchers undertaking the work in the participating NHS trusts. NET therapists will be interviewed by trainee clinical psychologist MG.

Data will be analysed using Braun and Clarke's thematic analysis [58] with the steps of (1) *Familiarization with the data*; (2) *Coding;* (3) *Generating themes*; (4) *Developing and reviewing themes*; (5) *Refining, defining, and naming themes* and (6) *Writing the report,* also incorporating consultation with peer interviewers (MB and ZK) and member checks using both single and synthesised analysed data [S1] in stages (3) and (4) respectively as described below.

The two trainee clinical psychologists AP and MG will complete steps (1) (2) and (3), with service user interviews being analysed first to privilege lived experience voices. Peer researchers AM and ZK will then review the generated themes for 25% of service user interviews. Feedback will be incorporated and generated themes amended as needed. Next, *member checks of single participant data* with 25% of service user participants and clinicians will be conducted. A synthesised summary of emerging themes with illustrative quotations of the participant's own interview will be shared with participants who gave consent. They will be asked if the emerging themes match their experiences and if they want to change/add anything. Following this, the two trainee clinical psychologists AP and MG will complete step (4) and again repeat *member checks* with service user and clinician participants but this time presenting synthesised data across the full data set with a thematic map; and they then will also consult with the NETp co-production group, which include lived experience researchers AM and KZ, as well as PPI lead NL, and three additional members with lived experience. Finally, steps (5) and (6) will be completed by AP and MG refining themes taking into account the learning from lived experience consultation and member checks conducted in the previous stage. The PI (MFA) and the PPI lead (NL) will offer supervision on all aspects of the analysis.

A critical realist perspective will be adopted and researcher reflexivity promoted [S2, S3] Member checking and consultation with lived experience researchers is therefore used to validate descriptive and interpretive data and to aid reflexivity. Within critical realism, the goal of member checking is not to establish truth or consensus as would be under a positivist epistemology but is instead to refine understanding by ensuring lived experience has been accurately captured, identifying gaps in researcher's perception.

Additional actions taken to enhance validity and reflexivity included bracketing [S4,S5] where researchers leading on the qualitative interviews (AP and MG) will engage in interviews and reflexive journaling to increase self-awareness of their own assumptions, theories and previous experiences of the phenomenon.

Supplementary material additional references:

S1 Birt L, Scott S, Cavers D, Campbell C, Walter F. Member Checking: A Tool to Enhance Trustworthiness or Merely a Nod to Validation? Qual Health Res. 2016 Nov;26(13):1802-1811. doi: 10.1177/1049732316654870. Epub 2016 Jul 10. PMID: 27340178.

S2 Braun, V., & Clarke, V. (2021). One size fits all? What counts as quality practice in (reflexive) thematic analysis?. *Qualitative research in psychology*, *18*(3), 328-352.

S3 Wiltshire, Gareth, and Noora Ronkainen. 2021. "A Realist Approach to Thematic Analysis: Making Sense of Qualitative Data Through Experiential, Inferential and Dispositional Themes." Journal of Critical Realism, March, 1–22. doi:10.1080/14767430.2021.1894909.

S4 Creswell, J. W., & Miller, D. L. (2000). Determining validity in qualitative inquiry. *Theory into practice*, *39*(3), 124-130.

S5 Tufford, L., & Newman, P. (2012). Bracketing in qualitative research. *Qualitative social work*, *11*(1), 80-96.
