# Supplementary material for: Narrative exposure therapy in early intervention in psychosis services (NETp): protocol of a multi-site feasibility randomised controlled trial study
Source: BMJ Open. 2026 Jun 22;16(6):e121914. doi: 10.1136/bmjopen-2026-121914 (PMC13288681; doi:10.1136/bmjopen-2026-121914)
Supplement: Supplementary file 2 [file bmjopen-2026-121914.reviewer_comments.pdf]

PEER REVIEW HISTORY

BMJ Open publishes all reviews undertaken for accepted manuscripts. Reviewers are asked to complete a checklist review form and are provided with free text boxes to elaborate on their assessment. These free text comments are reproduced below.

ARTICLE DETAILS

Title (Provisional)

Narrative Exposure Therapy in Early Intervention in Psychosis Services (NETp): Protocol of a multi-site feasiblity randomised Control trial study

Authors

Fornells-Ambrojo, Miriam; Plaistow, James; Hardy, Amy; Martin, Peter; Lyons, Natasha; Khan, Zarina; Mberi, Adeliede; Morris, Tiyi; Johnson, Sonia

---

This article was not externally reviewed at BMJ Open. Protocols that have been independently assessed prior to submission to BMJ Open are usually fast-tracked to publication on the grounds that further substantial changes will not be possible. This independent assessment will usually be external, independent review for both a substantial grant award from a non-commercial or government funder and ethics approval.
